# Physiologically Based Pharmacokinetic Modeling for Predicting Drug Levels After Bariatric Surgery: Vardenafil Exposure Before vs. After Gastric Sleeve/Bypass

**DOI:** 10.3390/biom15070975

**Published:** 2025-07-07

**Authors:** Daniel Porat, Oleg Dukhno, Sandra Cvijić, Arik Dahan

**Affiliations:** 1 Department of Clinical Pharmacology, School of Pharmacy, Faculty of Health Sciences, Ben-Gurion University of the Negev, Beer-Sheva 8410101, Israel; poratdan@post.bgu.ac.il; 2 Department of Surgery B, Soroka University Medical Center, Beer-Sheva 8410101, Israel; dukhnoo@bgu.ac.il; 3 Department of Pharmaceutical Technology and Cosmetology, Faculty of Pharmacy, University of Belgrade, Vojvode Stepe 450, 11221 Belgrade, Serbia; sandra.cvijic@pharmacy.bg.ac.rs

**Keywords:** metabolic surgery, oral absorption, drug solubility/dissolution, PBPK simulations, erectile dysfunction, PDE5 inhibitors, GastroPlus^®^

## Abstract

Bariatric surgery involves major changes in the anatomy and physiology of the gastrointestinal tract, which may alter oral drug bioavailability and efficacy. Phosphodiesterase-5 inhibitor (PDE5i) drugs are the first-line treatment of erectile dysfunction, a condition associated with a higher BMI. In this paper, we examine the PDE5i vardenafil for possible post-bariatric changes in solubility/dissolution and absorption. Vardenafil solubility was determined in vitro, as well as ex vivo using aspirated gastric contents from patients prior to vs. following bariatric procedures. Dissolution was tested in vitro under unoperated stomach vs. post-gastric sleeve/bypass conditions. Lastly, the gathered solubility/dissolution data were used to produce an in silico physiologically based pharmacokinetic (PBPK) model (GastroPlus^®^), where gastric volume, pH, and transit time, as well as proximal GI bypass (when relevant) were all adjusted for, evaluating vardenafil dissolution, gastrointestinal compartmental absorption, and pharmacokinetics before vs. after different bariatric procedures. pH-dependent solubility was demonstrated for vardenafil with low (pH 7) vs. high solubility (pH 1–5), which was confirmed ex vivo. The impaired dissolution of all vardenafil doses under post-gastric bypass conditions was demonstrated, contrary to complete (100%) dissolution under pre-surgery and post-sleeve gastrectomy conditions. Compared to unoperated individuals, PBPK simulations revealed altered pharmacokinetics post-gastric bypass (but not after sleeve gastrectomy), with 30% lower peak plasma concentration (C_max_) and 40% longer time to C_max_ (T_max_). Complete absorption after gastric bypass is predicted for vardenafil, which is attributable to significant absorption from the large intestine. The biopharmaceutics and PBPK analysis indicate that vardenafil may be similarly effective after sleeve gastrectomy as before the procedure. However, results after gastric bypass question the effectiveness of this PDE5i. Specifically, vardenafil’s onset of action might be delayed and unpredictable, negatively affecting the practicality of the intended use.

## 1. Introduction

The prevalence of obesity has substantially risen over the past few decades, reaching pandemic dimensions and becoming a huge global burden because of its association with some of the most prevalent chronic diseases, including diabetes, hypertension, and dyslipidemia [1]. Additionally, obesity is closely linked to erectile dysfunction (ED), both directly and indirectly. Among males who are overweight, ED is more common compared to those with a BMI under 25 kg/m^2^ and is more prevalent still in men with obesity [2]. Underlying pathophysiological and psychological factors can explain this association. Some of these factors include vascular problems, low testosterone, insulin resistance and type 2 diabetes, mental health and self-esteem, as well as chronic inflammation. (i) Vascular problems: Obesity increases the risk of high blood pressure, high cholesterol, and atherosclerosis. These conditions restrict blood flow, which can directly cause ED. (ii) Low testosterone: Men with obesity have lower levels of testosterone, which affects libido, mood and erectile function. (iii) Insulin resistance: Obesity is a major cause of type 2 diabetes, and men with diabetes are more likely to have ED. (iv) Mental health and self-esteem: Obesity is linked to depression, anxiety, and low self-image, all of which contribute to or worsen ED. (v) Chronic inflammation: Obesity triggers low-grade systemic inflammation, which disrupts nitric oxide (NO) signaling, critical for blood vessel relaxation during an erection [3].

Despite much evolved pharmacotherapeutic options for weight loss, bariatric surgery remains the most effective treatment option for severe obesity. Recently, eligibility for bariatric surgery has been updated to include patients with a BMI of 35 kg/m^2^ or above, regardless of obesity-related conditions, as well as BMI above 30 kg/m^2^ with a comorbidity. The success rate of surgery is high, allowing for longstanding weight reduction along with comorbidity remission, in particular type 2 diabetes. Different bariatric procedures are performed, with the most prevalent being the sleeve gastrectomy (SG), Roux-en-Y gastric bypass (RYGB), and the newer one-anastomosis gastric bypass (OAGB). After bariatric surgery, better sexual satisfaction and erectile function are described [4], attributable to improved blood flow and vascular health, hormonal balance, remission of underlying conditions [5,6], and a psychological boost [7,8]. In some cases, improvements are limited or absent due to persistent psychological factors, nerve damage from diabetes, and other reasons.

Phosphodiesterase-5 inhibitors (PDE5is) the most commonly used treatments for ED and work for 70–80% of men. Besides being highly effective, they are also non-invasive, convenient, fast acting, reversible and have minimal serious side effects [9,10]. The PDE5 enzyme breaks down cyclic guanosine monophosphate (cGMP), causing the erection to subside. By inhibiting this enzyme, PDE5is prevent the degradation of cGMP, allowing for sustained blood flow to the penis, resulting in an erection that is easier to obtain and maintain [11]. These orally administered drugs are generally well absorbed from the gastrointestinal tract (GIT) and have high systemic bioavailability. Oral absorption is multi-factorial and depends upon various parameters involving the drug, the drug product (dosage form and excipients), as well as the GIT physiology [12,13,14,15].

Bariatric surgery modifies the anatomy and physiology of the GIT dramatically. This may have a significant impact on all liberation, absorption, distribution, metabolism, and excretion (LADME) pharmacokinetic processes [14,16]. The gastrectomy component of the surgery involves a great decrease in functional stomach volume, alongside alterations in acid secretion and gastric motility [17,18]. These variables affect the drug release from the dosage form, a prerequisite for oral drug absorption [19,20]. The physicochemical parameters of the specific drug in question, including the dose, water solubility, and the presence of acidic/basic moieties, may aid in predicting the likelihood of absorption and bioavailability alterations after bariatric surgery [21,22,23].

**Figure 1:**
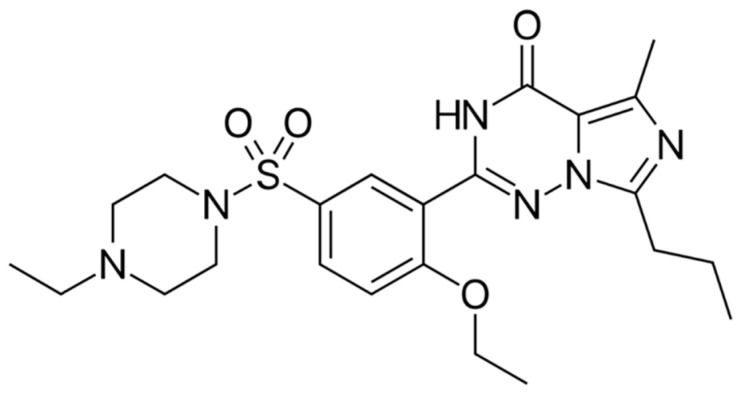
The molecular structure of vardenafil.

Sildenafil, vardenafil, and tadalafil are the three most commonly prescribed PDE5is. A recent study of sildenafil pre- vs. post-bariatric disposition was the first to investigate PDE5i treatment in the context of bariatric surgery [24]. Chemically, vardenafil is a weak base [25,26] (Figure 1) and thus may be prone to hampered dissolution and bioavailability after surgery. Physiologically based pharmacokinetic (PBPK) models are mathematical representations integrating drug-related and biological/physiological (system-related) parameters that can simulate the physiological drug behavior in tissues and blood over time in a virtual population [27]. Knowledge of the anatomical properties of the body and the mechanisms involved is the foundation of this approach [28]. PBPK models employ abundant information from various sources, including drug characteristics and physiological and biological parameters. PBPK has become indispensable for human PK evaluation from nonclinical data and is widely applied for drug discovery and development. PBPK methods also allow the extrapolation of the PK behavior of drugs regarding the route of administration, the dose, and population traits, including sex, BMI, and disease status [29]. A PBPK model comprises compartments that directly and realistically correspond to the body organs and tissues and connected by the cardiovascular and lymphatic systems. Integrating physiological parameters with in vitro and in vivo LADME data allows for generating a quantitative basis for extrapolating to different species or populations and predicting the impact of drug- and system-dependent features on drug exposure [30,31]. The gastrointestinal alterations following bariatric surgery are an additional challenge for the PBPK model application in estimating and simulating the PK changes under multiple conditions related to bariatric surgery. Nonetheless, the models integrate all available knowledge on known changes in the gastrointestinal (GI) tract following bariatric surgery. They can reflect the drug release from the dosage form and its dissolution, precipitation, and absorption along the GI tract under post-bariatric conditions [27]. Since each drug is unique in its physicochemical and pharmacokinetic properties and thus may be affected differently by the bariatric procedure, there is a need for extensive research in this field. Under these circumstances, in silico PBPK models, a relatively newly applied tool for the rapid evaluation of post-bariatric drug PK and its effects [24,27,32,33,34,35,36,37], assist with dosage recommendation and help achieve treatment goals amid the lack of clinical observations.

In this work, we study the in vitro/ex vivo solubility/dissolution, as well as the in silico compartmental absorption and overall pharmacokinetics (PK) and bioavailability of vardenafil in the setting of different bariatric procedures, analyzing the possible implications on post-bariatric PDE5i treatment.

## 2. Methods

### 2.1. Materials

Vardenafil hydrochloride powder (Angene International Limited, London, UK) was used in the solubility experiments. Vardenafil hydrochloride (Levitra^®^, Bayer Pharma AG, Leverkusen, Germany; batch no BXJ98N1) 20 mg tablets were used in the dissolution studies. The following materials were purchased from Sigma-Aldrich (Chemie GmbH, Steinheim, Germany) to be used in buffer preparation: maleic acid, acetic acid, monobasic sodium phosphate, sodium chloride, hydrochloric acid, and sodium hydroxide. UPLC-grade water and acetonitrile were purchased from Bio-Lab Ltd. (Ashkelon, Israel).

### 2.2. In Vitro Solubility

The equilibrium solubility of vardenafil was determined by the previously described shake-flask method [38,39]. Four different medium pHs were used: pH 1 and pH 3 (0.2 M maleic acid), pH 5 (0.2 M acetic acid), and pH 7 (0.2 M dihydrogen phosphate). Quadruplet (n = 4) samples of each buffer pH were prepared with drug powder added in excess, until saturation, to vials with 500 µL medium solution. Vial incubation was at 37 °C, with 200 rpm sample shaking for 24 h (Orbital Shaker Incubator, MRC Laboratory Instruments, Holon, Israel). Sample contents were then transferred to Eppendorf tubes for a 10 min centrifugation (Centrifuge 5430 R, Eppendorf^®^, Hamburg, Germany) at 20,817 rcf and 37 °C. Obtained supernatants were diluted as needed and immediately analyzed by UPLC-PDA (Table 1).

### 2.3. Ex Vivo Solubility

The equilibrium solubility of vardenafil in gastric content was also tested using the shake-flask method mentioned above, which followed a procedure that had been recently described [24]. In short, excess drug was added to glass vials containing samples of gastric content aspirated from three patients (1 SG, 1 RYGB, and 1 OAGB) during bariatric procedure time. Aspiration was performed through the nasogastric tube at three time points: before, immediately after , and 24 h post-surgery. Study protocol approval was given by the Ben-Gurion University School of Medicine institutional review board (institutional board request number 0248-18-SOR), and informed consent was obtained from all the participants prior to surgery. Following gastric content collection, pH was immediately measured and recorded. Samples were then vortexed, and 2 mL of fluid was extracted for centrifugation; supernatant fluid of 400 µL was used in the solubility experiment [34,40]. As vardenafil hydrochloride powder modified the pH of the post-bariatric gastric fluid, pH was adjusted with a small volume pH 7 buffer, as necessary.

### 2.4. In Vitro Dissolution

The dissolution of whole tablet vardenafil (20 mg) products was studied in three different conditions, as previously described [24] (n = 4 for each condition): (1) pH 1 maleic acid, 250 mL medium (pre-surgery conditions), with the USP dissolution apparatus II (Premiere 5100, Distek^®^, North Brunswick, NJ, USA), at 100 rpm paddle rotation [41,42]; (2) pH 5 acetic acid (post-SG conditions); and (3) pH 7 dihydrogen phosphate (post-OAGB conditions), both using 50 mL medium inside a 50 mL round-bottom flask in a water bath (37 °C) with mini-paddle (Cole Parmer^®^, Montreal, QC, Canada) rotating at 153 rpm. This rotation velocity was calculated, addressing the following parameters: the paddle size (*D*), volume of fluid (*V*), and agitation strength (*ε*) [43], as follows:

$$\frac{\varepsilon_{long}}{\varepsilon_{short}}=\frac{\frac{{RPM}_{long}^{3}\;\times \;D_{long}^{5}}{V_{long}}}{\frac{{RPM}_{short}^{3}\;\times \;D_{short}^{5}}{V_{short}}}$$

These adjusted experimental conditions mimic pre- vs. post-bariatric intragastric dissolution conditions, including the pH, temperature, fluid volume, and contractility force, and were thus preferred over standard, compendial dissolution methods [44,45]. Next, 300 µL samples were drawn for 90 min at 10 different time points and then filtered prior to ultra-performance liquid chromatography (UPLC) analysis (Table 1).

### 2.5. Analytic Method

Sample analysis was performed on a UPLC equipped with the Waters Acquity H-Class system and a PDA detector and controlled by Empower software. The analytic method used for vardenafil quantification is summarized in Table 1. Ambient samples and column temperatures were used. The calibration curve of each pH in relevant drug concentration ranges was linear (R^2^ = 0.999). Drug stability over the experimental course was verified.

### 2.6. Physiologically Based Pharmacokinetic (PBPK) Simulations

Drug-specific PBPK models were created to predict the peroral drug dissolution, absorption, and disposition based on its physicochemical and PK properties, using GastroPlus^®^ (version 9.8.3012; Simulations Plus Inc., Lancaster, CA, USA), aligned with parameters of specific human GIT physiological characteristics. These physiological values were integrated into the software-specific Advanced Compartmental Absorption and Transit (ACAT) model of the GIT and applied in a series of differential equations to simulate, following oral administration, the drug’s dynamic disposition [46,47]. For a healthy human representative in the fasted state, physiological parameters were kept at the software default values, except for small intestine and colon decreased percent fluid volumes, from the default 40% and 10% to 23% and 0.5% [48], respectively, accounting for the much smaller GI volumes in vivo [49,50,51]. Accounting for the altered physiological conditions after bariatric surgery, the ACAT model parameters were manually adjusted as follows: gastric volume was reduced from default 50 to 10 mL, consistent with the 80% reduction in gastric volume [52,53], and stomach transit time was reduced from default 0.25 to 0.12 h [54,55]. Simulations were also run for post-OAGB patients, accounting for the bypassing of the duodenum and jejunum [53]. Additionally, stomach pH was changed from pH 1.3 (default) to pH 5.0 (post-SG) and pH 7.0 (post-OAGB), accounting for the post-bariatric gastric pH increase, potentially affecting drug dissolution and absorption [14,17,56,57]. A summary of the GIT alterations in post-bariatric patients included in the models is provided in Table 2.

Input values regarding properties for vardenafil and their sources are summarized in Table 3. Values were either obtained from the literature, in silico predictions (ADMET Predictor^®^ module, version 10.4.0.0; Simulations Plus Inc., Lancaster, CA, USA) based on the drug’s molecular structure, or experimentally determined. pH-dependent solubility data were input as an .spd file and used to fit the solubility factors in the solubility model. Drug dissolution along the GIT was calculated using the software default Johnson equation [58] based on the drug’s solubility and particle size. With the lack of data on commercial product drug particle size, 100 µm was chosen as an approximate value for all simulations. Regarding poor aqueous solubility, bile salts’ effect on drug solubility/dissolution was also accounted for. The drug precipitation time was set as the default 900 s. Although this parameter may affect the disposition of basic compounds, parameter sensitivity analysis demonstrated that variations in the 10-fold range of the default input value will not affect the rate and extent of vardenafil absorption. The distribution and elimination PK parameters of vardenafil were based on available data of in vivo plasma concentration over time following IV and PO drug administration, using the software-integrated PKPlus^TM^ module and optimized further while keeping the final values within the reported literature range (Table 3).

The predictive power of the designed model was evaluated by comparing the software-predicted with the in vivo-observed values for the area under the plasma concentration–time curve (AUC_0−inf_), maximum plasma concentration (C_max_) and time to reach C_max_ (T_max_), for 10 mg and 20 mg single oral doses of vardenafil administered to healthy human subjects in the fasted state. The predicted values referred to a healthy human participant, and the in vivo values were digitized (DigIt software, version 1.0.4; Simulations Plus, Inc., Lancaster, CA, USA) from mean profiles observed in a published clinical study [59]. Due to the lack of demographic data for the clinical study, an average body weight of 70 kg (software default value) was used for the simulations. A comparison was performed by calculating fold errors between observed and predicted data, whereat fold error represents the ratio between the predicted and observed values. According to the commonly applied criteria, for most drugs, a 2-fold error may be considered acceptable, while for drugs with, e.g., low variable PK, tighter limits in a 1.5-fold range may be more appropriate, whereas for drugs with high PK variability, a less stringent 2.5-fold dependence can apply [60,61]. In addition, to assess the observed and predicted values’ linearity, the coefficient of determination (R^2^) was used.

**Table 3:** Input parameters for vardenafil PBPK model.

	Vardenafil	
Parameter	Value	Source/Ref
Molecular weight (g/mol)	488.61	/
LogP/LogD	1.97	predicted using ADMET Predictor^®^ module
Solubility at 37 °C (mg/mL)	30 (pH 1); 30 (pH 3); 27.4 (pH 5); 0.05 (pH 7)	Experimental
pKa (s)	4.24 (base); 7.76 (base); 8.68 (acid)	predicted using ADMET Predictor^®^ module
Solubility factors	720.8; 577.0; 393.9	fitted by the software integrated option (based on the pH-solubility profile)
Human effective permeability, Peff (cm/s)	5.00 × 10^−4^	optimized ^a^
Diffusion coefficient (cm^2^/s)	0.5913 × 10^−5^	software calculated (based on drug molecular weight)
Particle diameter (µm)	100	Approximated
Mean precipitation time (s)	900	software default
Drug dose (mg) (dosage form)	10 ^b^, 20 (tablet)	/
Volume of fluid taken with drug (mL)	250 (pre-surgery); 50 (post-surgery)	software default or decreased by 80% to comply with the decreased gastric volume [52] and limited volume of fluid the bariatric patient can ingest
Blood/plasma concentration ratio	0.83	predicted using ADMET Predictor^®^ module
Plasma fraction unbound (%)	5	[59,62,63]
First pass effect, FPE (%)	83	optimized to comply with the literature-reported values [62,64]
Clearance, CL (L/h/kg)	0.925	estimated using PKPlus^TM^ module, based on the in vivo data for IV and oral drug doses [59] and then optimized while keeping the optimized values within the range reported in the literature [62,63,64]
Volume of distribution, Vd (L/kg)	0.800	
Distribution constant k_12_ (1/h)	3.090	
Distribution constant k_21_ (1/h)	2.100	
Distribution constant k_13_ (1/h)	0.448	
Distribution constant k_31_ (1/h)	0.250	
Elimination half-life, t_1/2_ (h)	4.43	software calculated; complies with the reported data [62,64]

^a^ complies with literature data on enhanced permeability of vardenafil relative to sildenafil due to the presence of an extra methylene lipophilic group [65,66]; ^b^ solely for the model validation.

## 3. Results

### 3.1. In Vitro Solubility

**Figure 2:**
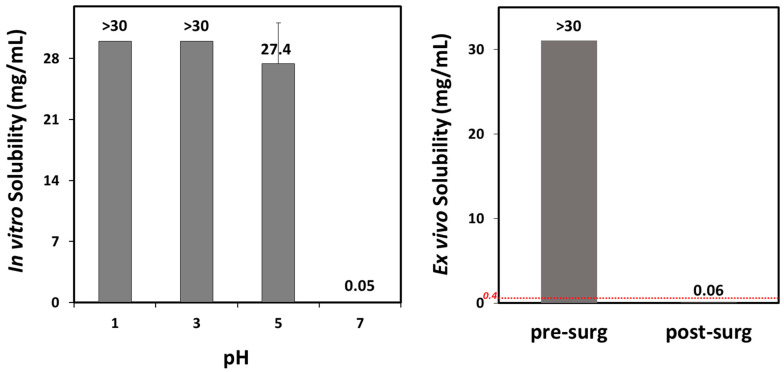
Vardenafil saturation solubility. Left panel: in vitro solubility as a function of pH. Data presented as mean (SD); n = 4 for each pH. Right panel: ex vivo solubility in intragastric aspirate samples from three patients before (left column; mean pH 2.0) vs. after (right column; mean pH 7.0) bariatric surgery. Dashed red line represents threshold solubility for complete post-bariatric dissolution of a 20 mg vardenafil dose. Data presented as mean (SD); n = 3 for each experimental group.

The solubility of vardenafil decreased with increasing pH. Consistent with its basic nature, solubility in pH 1 was over 30 mg/mL. However, at pH 7, vardenafil had low solubility of 0.05 mg/mL, an over 600-fold decrease in saturation solubility (Figure 2, left panel).

### 3.2. Ex Vivo Solubility

Ex vivo solubility in intragastric contents from patients undergoing bariatric surgery (patient characteristics are summarized in Table 4) was consistent with the in vitro solubility results showing significantly lower solubility for vardenafil (>500-fold) after vs. before the surgery (Figure 2, right panel). Upon the addition of excess vardenafil HCl powders, the intragastric fluid pH decreased, attributable to the HCl component; therefore, pH 7 buffer was added, as necessary, to adjust the pH to the values that were initially measured.

### 3.3. In Vitro Dissolution

**Figure 3:**
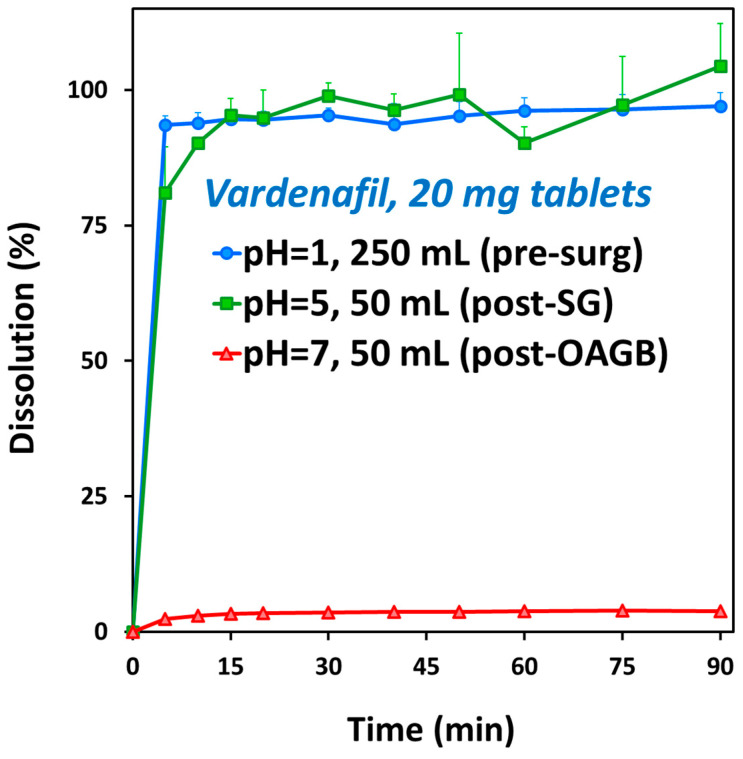
Vardenafil in vitro dissolution of commercially available Levitra^®^ (vardenafil) 20 mg tablets. 250 mL at pH 1 medium (blue circles) represent pre-surgery gastric conditions; 50 mL at pH 5 medium (green squares) represent post-SG scenario; 50 mL at pH 7 medium (red triangles) represent post-OAGB gastric scenario. Average (SD); n = 4 for each experimental group.

The dissolution of vardenafil was complete (100%) in pre-surgery, pH 1 conditions, as well as in post-SG, pH 5 conditions. Meanwhile, in post-OAGB, pH 7 conditions, the dissolution of vardenafil was markedly impaired, with only 4% dissolved from the drug products (Figure 3). In other words, after surgery, the dissolution of vardenafil largely depends on the pH.

### 3.4. Physiologically Based Pharmacokinetic (PBPK) Simulations

The simulated PK parameters (C_max_, T_max_, AUC_0−inf_) for different doses of oral vardenafil agree with the mean data obtained in clinical trials (Table 5), revealing good prediction power of the present PBPK model. According to the calculated R^2^ value (Table 5), the simulated and observed data correlate. The calculated fold errors for the tested vardenafil oral doses (10 mg and 20 mg) comply with the 1.25-fold criterion (range between 0.80 and 1.25), which equals the criterion applied for bioequivalence studies. The designed drug-specific PBPK model was finally used to simulate vardenafil in vivo dissolution and absorption in post-bariatric vs. healthy subjects and to mechanistically determine the joint effect of compound physicochemical and physiological factors on the PK and bioavailability of vardenafil.

**Figure 4:**
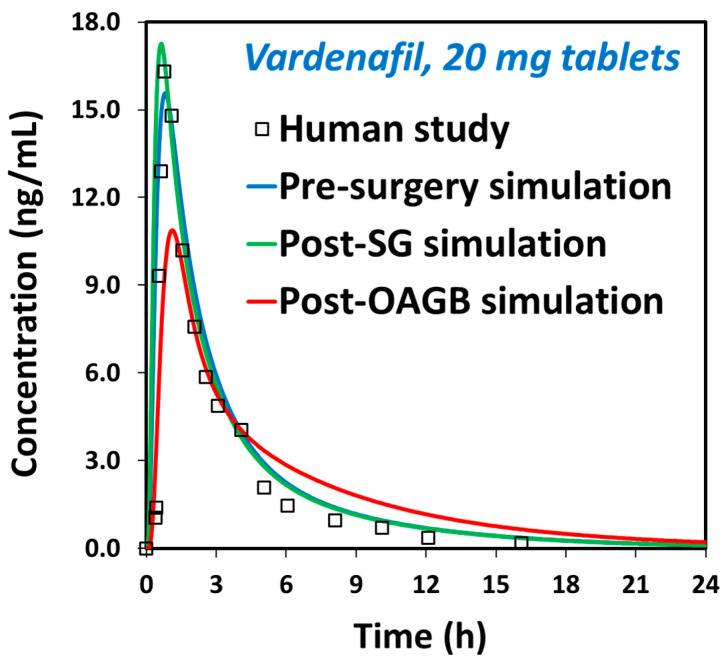
The predicted plasma concentration–time profiles under pre-surgery, post-SG, and post-OAGB scenarios for vardenafil 20 mg. Empty squares represent the mean values obtained in human studies [59].

**Figure 5:**
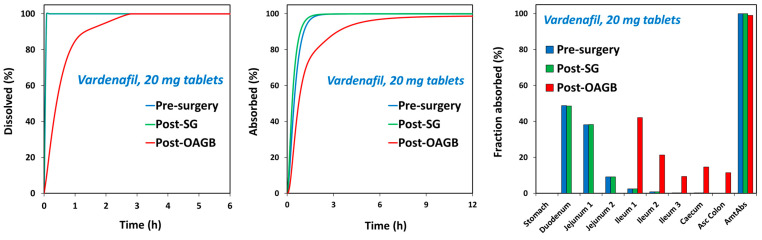
The simulated in vivo dissolution (left panel) and absorption (middle panel) predicted profiles, along with the simulated GI tract regional absorption (right panel) predicted for vardenafil 20 mg tablets under pre-surgery, post-SG, and post-OAGB scenarios.

The effect of post-bariatric GI physiology changes on vardenafil indicates certain changes in C_max_ and T_max_ values, but with no alterations in the overall drug exposure (Figure 4; Table 6). Due to relatively high vardenafil solubility at pH up to 5 (Figure 2), the complete drug dose is dissolved in the post-SG state (Figure 3 and Figure 5, left panel), and since gastric transit time in the post-surgery state is decreased, vardenafil may exhibit slightly higher C_max_ and shorter T_max_ in the post-SG vs. pre-surgery state (Figure 4; Table 6). Slightly faster vardenafil absorption in the post-SG state is also visible in the simulated absorption profile (Figure 5, middle panel). In the post-OAGB state, the proximal intestine is bypassed, resulting in delayed drug absorption (Figure 5, middle panel). Also, slower drug dissolution in the case of the elevated gastric pH 7, characteristic of OAGB procedures [17], leads to a 30% C_max_ decrease (Table 6). However, high drug permeability allows for increased vardenafil absorption in the more distal intestine (Figure 5, right panel). Moreover, unmaterialized absorption in the bypassed proximal intestine leads to less first-pass metabolism in the liver, as indicated by a 36% decrease in maximum vardenafil liver concentration in post-OAGB patients compared to the pre-surgery-predicted value. This observation complies with the reported information [67]. According to the simulation results, the net effect of these changes is that vardenafil oral absorption remains unaffected by bariatric surgery.

## 4. Discussion

In this work, we show that the dissolution of vardenafil is potentially impaired after bariatric surgery. This is due to decreased stomach volume and, in particular, increased intragastric pH. For vardenafil, post-operative gastric pH is an important factor attributable to its weak basicity [68], so highly increased intragastric pH, especially after bypass procedures, could produce a substantial decrease in solubility [17]. Indeed, low solubility is observed in stomach contents of high pH (~7), obtained from patients after different bariatric procedures. Yet, overall absorption is predicted to be complete through high absorption from distal intestinal segments. Regardless, vardenafil is predicted to have lower and delayed C_max_, which may significantly affect the drug’s onset of action. Similar results were found for the structurally closely related PDE5i sildenafil. It could be expected that the PK of vardenafil, as opposed to sildenafil, may not be altered at all; this is due to the much lower maximal single unit dose of vardenafil vs. sildenafil, theoretically allowing for rapid dissolution and absorption from more proximal intestinal segments. However, our in silico PBPK results reveal that the effects of OAGB on the PK of vardenafil highly resembles that of sildenafil. Consequently, after half an hour post-dosing, the time the drug should have a pharmacological effect in most cases, plasma concentrations in the post-OAGB scenario are markedly low (Figure 4). With a pKa of ~7, basic drugs such as vardenafil and sildenafil may be sufficiently dissolved under the dynamic “sink-like” physiologic conditions, even in the limited volume, operated stomach after gastric bypass surgeries. These conditions are properly portrayed in the PBPK simulations, as opposed to the non-sink characteristics of in vitro dissolution tests, highlighting the great contribution of these advanced in silico tools in the attempt to characterize post-bariatric PK behavior of drugs. Physiologically, despite much higher gastric pH, the percent ionization of vardenafil molecules is large enough to overcome these unfavorable GI conditions, maintaining complete dose dissolution, even if delayed (Figure 5, left panel). For weaker bases, which are effectively completely unionized under high post-bariatric pH, including lamotrigine, loratadine and etoricoxib, this was not the case [33,34,40].

As mentioned, obesity and ED are associated, and while sexual function may improve after bariatric surgery, there are limited data on post-bariatric changes in PDE5i use [69]. After the surgery, many patients may still depend on PDE5is due to the reasons stated above [24]. Slower absorption (as also shown for sildenafil) following gastric bypass surgery likely means that the use of vardenafil shortly before intercourse, as typically instructed, may not be as effective as in unoperated individuals. Taking this drug at an earlier time, as may be required after gastric bypass, is inconvenient and even impractical. Delayed absorption may also produce high unpredictability regarding the onset and duration of action to the point of compromised reliability of the treatment. Indeed, while in most cases, the extent of drug absorption is the main focus, in the case of PDE5is, the absorption rate appears to be just as important. Other mechanisms affected by bariatric surgery may well be involved in the PK of vardenafil but are aspects not covered in this work [70,71]. It should be mentioned, though, that following weight loss, CYP3A activity is increased [72,73], and since vardenafil is predominantly metabolized by CYP3A4, its PK may be affected by bariatric surgery to an even larger extent than predicted in this work [73,74].

## 5. Conclusions

In conclusion, vardenafil drug products used as a rapid-onset pill may not be quite as efficacious and reliable among patients after gastric bypass surgery. A post-bariatric human study is called for to prove that the pharmacological success of vardenafil is not limited by the surgery; in the meantime, clinical monitoring may be the best bet for these patients.

## Figures and Tables

**Table 1:** UPLC-PDA analytic method for vardenafil solubility/dissolution studies.

Drug	Column	Mobile Phase	Flow Rate (mL/min)	Injection Volume (µL)	Total Run Time (min)	Retention Time (min)	Detection Wavelength (nm)
Vardenafil	Waters XBridge C8, 3.5 µm, 4.6 × 150 mm	Water: Acetonitrile (+0.1% trifluoroacetic acid), 90:10 to 15:85 (*v*/*v*), gradient	1.0	20	5.0	3.6	245

**Table 2:** Changes in GIT physiology between pre- and post-bariatric patients included in the PBPK models. OAGB, one-anastomosis gastric bypass; SG, sleeve gastrectomy.

Parameter/Property	Healthy	SG	OAGB
Stomach volume	50 mL	10 mL	10 mL
Volume of fluid taken with drug (average)	250 mL	50 mL	50 mL
Stomach pH	1.3	5.0	7.0
Stomach transit time (fasted)	0.25 h	0.12 h	0.12 h
Intestinal segments available for absorption	default	default	bypassed duodenum and jejunum

**Table 4:** Patient characteristics including measured intragastric pH before vs. one day after bariatric surgery. RYGB, Roux-en-Y gastric bypass; OAGB, one-anastomosis gastric bypass; SG, sleeve gastrectomy.

Patient	Age	Gender	BMI	Procedure	Pre-Surg pH	Post-Surg pH
1	49	Male	43	RYGB	2.7	6.8
2	51	Female	39	OAGB	1.5	7.2
3	25	Male	71	SG	2.0	7.0

**Table 5:** Comparison of the simulated and observed pharmacokinetic parameters for vardenafil.

10 mg	Observed ^a^	Predicted	Fold Error	R^2^ Value
C_max_ (ng/mL)	7.20	7.78	1.08	0.9750
T_max_ (h)	0.95	0.80	0.84	
AUC_0−inf_ (ng h/mL)	27.29	26.25	0.96	
20 mg	Observed ^a^	Predicted	Fold error	
C_max_ (ng/mL)	16.30	15.57	0.95	
T_max_ (h)	0.78	0.80	1.03	
AUC_0−inf_ (ng h/mL)	41.86	52.51	1.25	

^a^ Estimated from the mean plasma concentration–time profiles.

**Table 6:** Percent decrease/increase ^a^ in the simulated values of pharmacokinetic parameters for vardenafil after bariatric surgery compared to pre-surgery state.

20 mg	Post-SG (Gastric pH = 5)	Post-OAGB (Gastric pH = 7)
C_max_	−10.85	30.12
T_max_	20.00	−40.00
AUC_0−inf_	0.00	0.93

^a^ Calculated as: (value_pre-surgery_ − value_post-surgery_) × 100/value_pre-surgery_.

## Data Availability

The original contributions presented in this study are included in the article. Further inquiries can be directed to the corresponding author.
